# The Retrograde Fractional Meeting at St. Louis

**Published:** 1904-08

**Authors:** 


### THE RETROGRADE FRACTIONAL MEETING AT ST. LOUIS.

In an article in this present issue, written some time previous to the meeting of a number of the members of the National Association of Dental Faculties at St. Louis, the views of the writer were given up to that time, but the meeting of the dissatisfied has now been held, and its action on July 18 is open to the support or condemnation of the dental profession. Twenty-seven colleges were represented out of a total of fifty-three members. How many of these had a legal right to sit there and vote is not known, as the number or names of colleges having resigned was refused when the request was made. This, as it stood, represented half the colleges connected with the Association.

No forced construction could make this body a meeting of the National Association of Faculties, but it was a special gathering of those who suffered defeat at Washington, D. C., in June, with a few additional interested observers.

It is difficult to treat of this meeting with that calm consideration due the serious results that may follow its deliberations. It marks one of the most important departures from a correct standard of dental education that has been experienced in fifty years. To treat, therefore, its retrograde action with complacency is impossible. It carries the dental profession in these United States back to a period in the sixth decade of the last century, when chaos reigned and every educational man's hand was against every other man, a condition not changed until the National Association of Faculties was organized in 1804. All that that organization has patiently and persistently worked for has been cast to the four winds, and unless there be a courageous effort made to withstand this sudden wave of commercialism, it is feared the last state will be found worse than the first.

That our readers may know exactly what was done by this retrograde faction, we give the following resolutions:

> *Resolved,* That the minimum time for dental teaching required by this Association to qualify students for examination for graduation shall be thirty weeks of six days each, in each of three separate academic years, exclusive of holidays. This resolution to take effect at once; and be it further
>
> "*Resolved,* That all rules, or parts of rules, in conflict with this resolution be and are hereby repealed."

That these resolutions were passed by and at a meeting convened without any semblance of authority under the constitution need not be stated to any one conversant with the rules and usages of the Faculties Association, but this article is for the enlightenment of the profession generally, and not especially for the members of that organization. That a body of men could come together and unitedly agree to violate all the rules in order to carry out their predetermined plan is beyond the comprehension of the writer. An organization containing such a membership is a rope of sand and unworthy the respect of the intelligent thinking portion of the dental profession.

The past year has witnessed three meetings called without authority, and each one naturally led to the succeeding as a wilful violation of the rules governing the organization. The following bear directly upon what shall constitute a quorum. It was not to be expected that such a body would pay much attention to the slight matter—to them—of a quorum.

> "Article IV. Two-thirds of the colleges belonging to this Association shall be necessary to constitute a quorum. In all matters not in conflict with Article VIII. of the constitution, a majority of the colleges belonging to this Association shall constitute a quorum."

Turning to Article VIII., it reads: "Any contemplated change involving the interests of the schools represented, or of the Association, shall require one year's notice before any action is taken." Compare this constitutional provision with the action at this St. Louis meeting, where the resolutions adopted directly affected the interests of all the schools, and should, in a properly regulated body, have been held over for action next year. But this body went farther than this and ordered that "all rules and parts of rules in conflict with these resolutions be and are hereby repealed." Lawlessness could go much farther, and the resolutions seemed to give great satisfaction.

There was one slight sign of sensitiveness to what the world might say, for this peculiar body passed a resolution that hereafter all proceedings should be held sacred and nothing should be permitted publication until the official sanction had been given. This spasm of sensitiveness was the result of the publication of the vote at Washington adopting the four-year rule. We are not surprised that such a resolution was passed, but if these educators expect that the profession will close its eyes and ears to their doings, or that this journal will cease to make note of their proceedings, they have misjudged the temper of the profession and the character of the editor of the INTERNATIONAL DENTAL JOURNAL.

The result of such work is not easy to determine, but if the history of dental education in this country counts for anything, we must expect that when these schools are a law unto themselves, there will be, as a result, a violent competition, that will end in the destruction of all the weaker schools and, in the end, the survival of the stronger. If this were all, it might be contemplated with serenity, but it means more than this,—a body of unqualified men forced into the dental profession unless these are halted at the portals of the State boards. What will be the action of these final arbiters of ability it is not difficult to determine. In those States lacking a law requiring four years they will eventually be passed into the ranks of the qualified.

It is probably true that all the dental schools will experience a period that will try them to the uttermost, but out of this chaotic condition, the writer feels assured, will come the golden period of stability in educational methods. The weeds in our educational garden will all have been dug up and cast into the ash-pits of civilization, and the refreshed garden will yield a thousand-fold more to the glory of the dental profession than at any period of the past.

Whether the Association of Faculties as originally organized will survive this blow remains to be seen. Confidence in it and its proceedings will be entirely shattered. It will require years to build up the confidence lost by the action at St. Louis. It is to be hoped that there will be a remnant, with courage unshaken, who will take up this work and leave those who passed that resolution at St. Louis to flounder in the mire of their own creating. The true Association of Faculties has stood for the highest in dental education, and by the help and encouragement of all that are loyal to the faith it will still work on through the twentieth century until the commercial taint in our profession is entirely eradicated.

