# Correction

**Published:** 1904-09

**Authors:** 


### CORRECTION.

In the last line on page 634 of last number the types made our editorial say, "The National Association of Dental Faculties was organized in 1804." It should have read 1884.

